# Supplementary material for: G-quadruplex stabilization provokes DNA breaks in human PKD1, revealing a second hit mechanism for ADPKD
Source: Nat Commun. 2025 Jan 2;16:121. doi: 10.1038/s41467-024-55684-y (PMC11696556; doi:10.1038/s41467-024-55684-y)
Supplement: Supplementary file 1 — Supplementary Information [file 41467_2024_55684_MOESM1_ESM.pdf]

Supplementary Figure 1.

Circular dichroism spectroscopy of G4-folded oligonucleotides from human *PKD1* G-rich introns. Sequences, guanine repeats underlined, were derived from IVS21; AGGGGAGGAGGGGAGGAGGGAGGAGGGGA, blue, IVS22; ATAAGGGAGGGGAAGGGGGATGAGGGGGATGA, red, and IVS42; GCTGCCGGGGCGGGGCCCTGCGAGGGGGCGGGACGCTG, green. Peaks around 260 nm and a dip at 240 nm are consistent with G4 DNA structures.

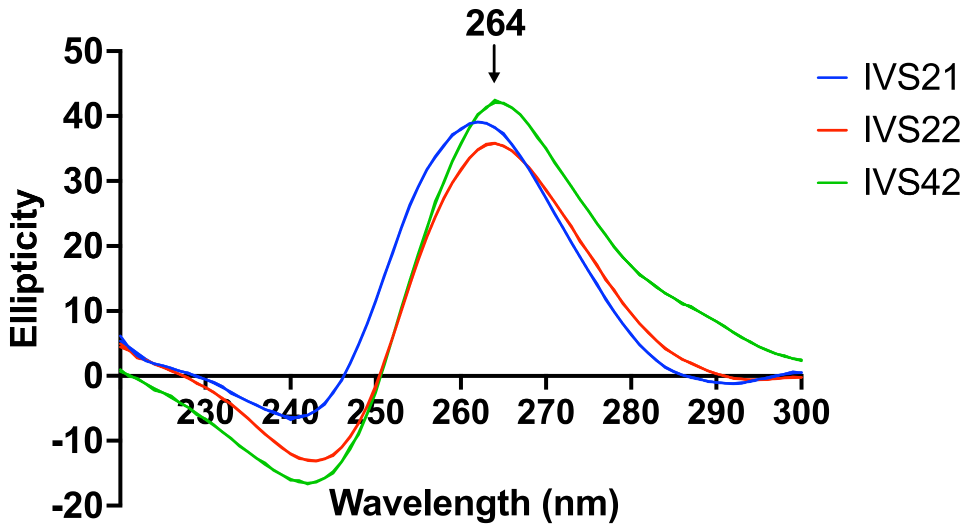

Supplementary Figure 2.
Immunolabeling of G4 quadruplex structures in HEK293T and ADPKD tissue. Panel A, immunofluorescent localization of G4 DNA structures in HEK293T nuclei with BG4 antibody (red) and secondary antibody only control (inset in A). Panels B and C, immunofluorescent localization of G4 DNA structures in human ADPKD tissue nuclei with BG4 antibody (green dots). Boxed region in B is enlarged in C to show numerous G4 DNA structures in the cystic epithelium nuclei. Panels D and E, immunofluorescent localization of G4 DNA in human ADPKD tissue nuclei with SG4 nanobody (green dots). Boxed region in D is enlarged in E to show numerous G4 DNA structures in the cystic epithelium nuclei (arrows). Panel F is human ADPKD tissue with a mutated control nanobody (SG4mut-R105A). Inset in F is enlargement of boxed region. Scale bars in microns, A=50, B=50, C=10, D=50, E=10, F=50, inset=10.

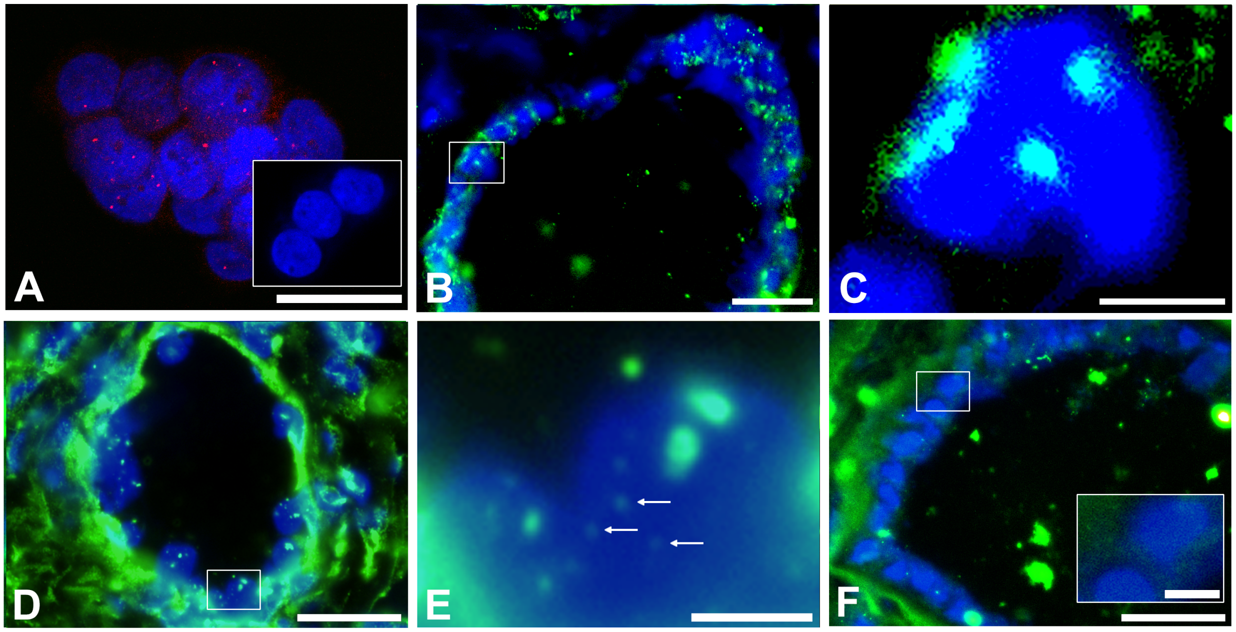

Supplementary Figure 3. Control showing that Phen-DC3 and CX-5461 ligands function in mIMCD3 cells. BG4-IPs show enrichment of the G4-rich mouse Sgamma3 region when Phen-DC3 is present, left, with no enrichment of m*Pkd1* observed. This was repeated with CX-5461, right, with similar results, indicating that both ligands are functional in mIMCD3. Mouse Sgamma3 qPCR used AGGGGACCTGGATAAGCCAT and GCTTCAGCTTCCCTGTAGCA primers. The m*Pkd1* amplicon used the same primers used to capture IVS21 (Fig. 4). Data are presented as mean +/- s.e.m. *n*=3 technical replicates for each experiment.

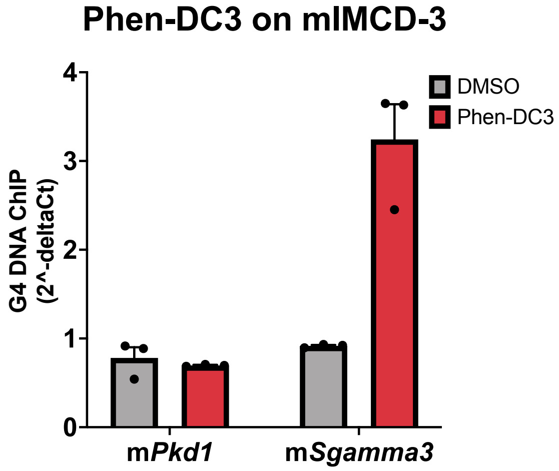

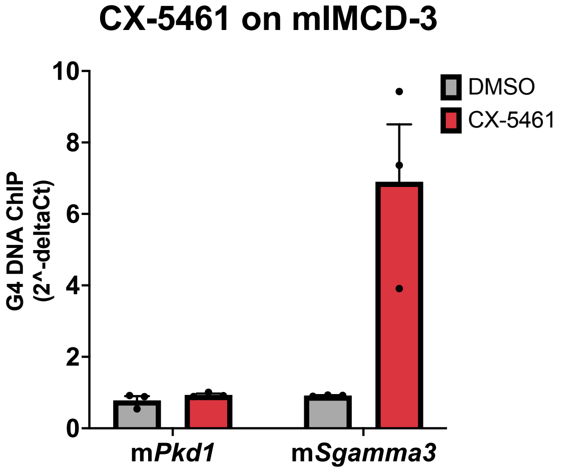

Supplementary Figure 4. Uncropped western blot with anti-PC-1 antibody on whole cell lysates of HEK293T treated with Phen-DC3 or DMSO for 1, 2, 7, or 14 days. Due to the size difference, beta-actin Western blot loading control was run at the same time but on another blot. Multiple bands are expected in whole cell extracts because polycystin-1 is proteolytically processed.

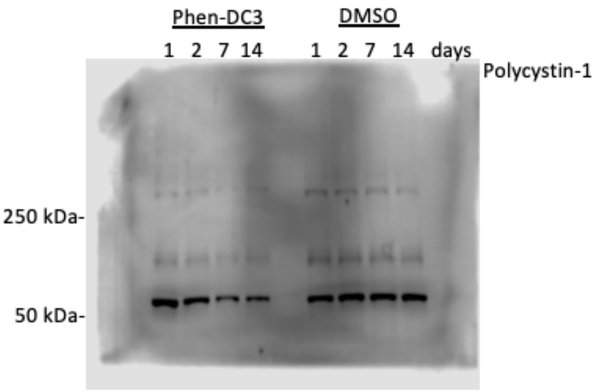

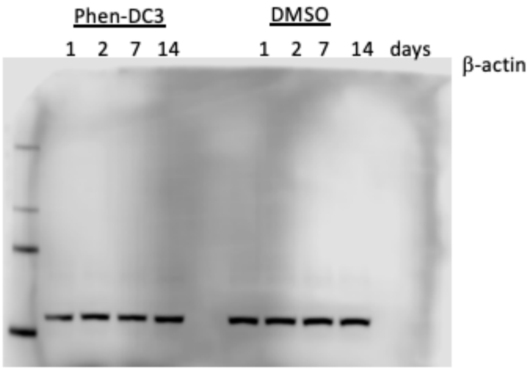

Supplementary Figure 5. Mapping of both G4 motifs and somatic exon mutations on h*PKD1*. Somatic mutations (•), regardless of type, were derived from prior whole exome and whole genome sequencing of individual renal cyst epithelia from APDKD patients [1,2] and are displayed below the gene. G4 motifs (+) are indicated above the gene. Select exons are indicated below the mutations as a reference.

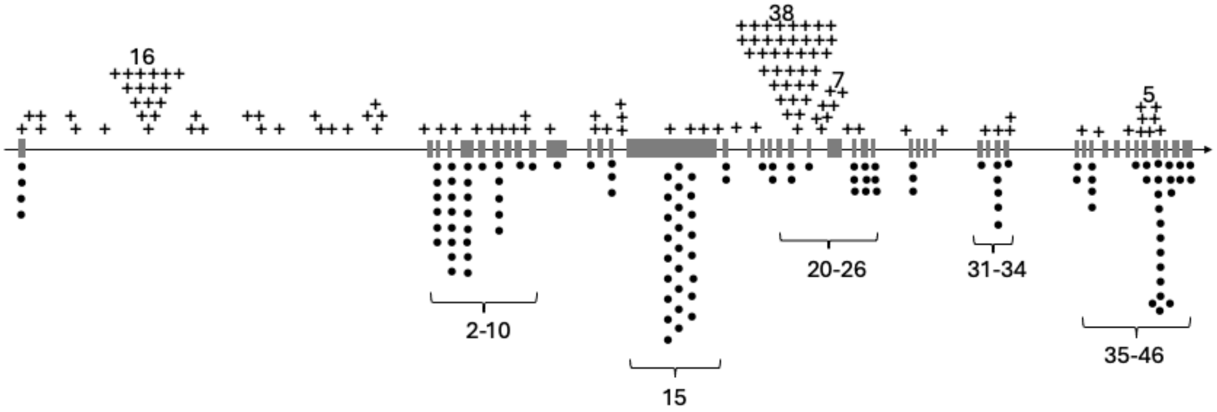

Supplementary Table 1. Oligonucleotides used for BG4 ChIP.

hIVS21	CCCCTCTGCTCGTAGGTCTT
	GCACTGACCCACAACACTGA
hIVS34	ATGTAGTCACGCACATAGTCAC
	TGCCCTCATGTGATGGTTATCT
mIVS21	CAGAAGCTGGAGGGCATGAT
	CCTCATTGAGCACTCGGGAA
mIVS37	TGGCACTTATGCCCACCAAT
	CCCATTGGGTATCCCAGCAC

Supplementary Table 2. Oligonucleotides used in gammaH2AX ChIP

h*PKD1*	CCCCTCTGCTCGTAGGTCTT
	GCACTGACCCACAACACTGA
h*PCNA*	TGAACCTTGTTTTGTAGGTAGTCA
	TGCATTTAGAGTCAAGACCCTTT
m*Pkd1*	CAGAAGCTGGAGGGCATGAT
	CCTCATTGAGCACTCGGGAA
m*Pcna*	AGATGTGCCCCTTGGTAAGA
	AGCAAATTATGGAGAATGGAGAGA

Supplementary Table 3. Oligonucleotides used for RT-qPCR

h*PKD1*	GGCCATCCTGCTCGTGTCTT
	GGTAGCGCCAGCGGAGAATA
hβ*actin*	TGGAGAAAATCTGGCACCACAC
	ATGGCTGGGGTGTTGAAGGT
m*Pkd1*	TGCGCCAGTGGTCTGTTTTT
	CCAGGACTCTGAAGGGCACA
mβ*actin*	GGCTGTATTCCCCTCCATCG
	CCAGTTGGTAACAATGCCATGT

Supplementary References

1. Zhang, Z. *et al.* Detection of PKD1 and PKD2 somatic variants in autosomal dominant polycystic kidney cyst epithelial cells by whole-genome sequencing. *Journal of the American Society of Nephrology* **32**, 3114–3129 (2021).
2. Tan, A. Y. *et al.* Somatic mutations in renal cyst epithelium in autosomal dominant polycystic kidney disease. *Journal of the American Society of Nephrology* **29**, 2139–2156 (2018).
