# Supplementary material for: G-quadruplex stabilization provokes DNA breaks in human PKD1, revealing a second hit mechanism for ADPKD
Source: Nat Commun. 2025 Jan 2;16:121. doi: 10.1038/s41467-024-55684-y (PMC11696556; doi:10.1038/s41467-024-55684-y)
Supplement: Supplementary file 3 — Description of Additional Supplementary Files [file 41467_2024_55684_MOESM3_ESM.pdf]

<u>Description of Additional Supplementary Files</u>

Supplementary Data 1. Sequences: DNAs used for sgRNA synthesis
