# Supplementary material for: G-quadruplex stabilization provokes DNA breaks in human PKD1, revealing a second hit mechanism for ADPKD
Source: Nat Commun. 2025 Jan 2;16:121. doi: 10.1038/s41467-024-55684-y (PMC11696556; doi:10.1038/s41467-024-55684-y)
Supplement: Supplementary file 5 — Reporting Summary [file 41467_2024_55684_MOESM5_ESM.pdf]

nature portfolio

Corresponding author(s): Erik Larson

Last updated by author(s): Nov 26, 2024

Reporting Summary

Nature Portfolio wishes to improve the reproducibility of the work that we publish. This form provides structure for consistency and transparency in reporting. For further information on Nature Portfolio policies, see our Editorial Policies and the Editorial Policy Checklist.

Statistics

For all statistical analyses, confirm that the following items are present in the figure legend, table legend, main text, or Methods section.

n/a	Confirmed	
☐	☒	The exact sample size (*n*) for each experimental group/condition, given as a discrete number and unit of measurement
☐	☒	A statement on whether measurements were taken from distinct samples or whether the same sample was measured repeatedly
☐	☒	The statistical test(s) used AND whether they are one- or two-sided<br>*Only common tests should be described solely by name; describe more complex techniques in the Methods section.*
☒	☐	A description of all covariates tested
☐	☒	A description of any assumptions or corrections, such as tests of normality and adjustment for multiple comparisons
☐	☒	A full description of the statistical parameters including central tendency (e.g. means) or other basic estimates (e.g. regression coefficient) AND variation (e.g. standard deviation) or associated estimates of uncertainty (e.g. confidence intervals)
☐	☒	For null hypothesis testing, the test statistic (e.g. *F*, *t*, *r*) with confidence intervals, effect sizes, degrees of freedom and *P* value noted<br>*Give P values as exact values whenever suitable.*
☒	☐	For Bayesian analysis, information on the choice of priors and Markov chain Monte Carlo settings
☒	☐	For hierarchical and complex designs, identification of the appropriate level for tests and full reporting of outcomes
☒	☐	Estimates of effect sizes (e.g. Cohen's *d*, Pearson's *r*), indicating how they were calculated

Our web collection on statistics for biologists contains articles on many of the points above.

Software and code

Policy information about availability of computer code

Data collection	A web-based software program was used to calculate G4 content, https://bioinformatics.ramapo.edu/QGRS/index.php. Endoquad was also referenced, http://endoquad.chenzxlab.cn/#/group-g4
Data analysis	Prism software Version 10.2.1 was used to perform one way and two way ANOVAs.

For manuscripts utilizing custom algorithms or software that are central to the research but not yet described in published literature, software must be made available to editors and reviewers. We strongly encourage code deposition in a community repository (e.g. GitHub). See the Nature Portfolio guidelines for submitting code & software for further information.

Data

Policy information about availability of data

All manuscripts must include a data availability statement. This statement should provide the following information, where applicable:

- Accession codes, unique identifiers, or web links for publicly available datasets
- A description of any restrictions on data availability
- For clinical datasets or third party data, please ensure that the statement adheres to our policy

All data and supporting information are available in the article and supplementary materials. A source data file is also included.

Research involving human participants, their data, or biological material

Policy information about studies with human participants or human data. See also policy information about sex, gender (identity/presentation), and sexual orientation and race, ethnicity and racism.

Reporting on sex and gender	male
Reporting on race, ethnicity, or other socially relevant groupings	unknown
Population characteristics	unkonwn
Recruitment	unkonwn
Ethics oversight	Use of de-identified tissues here was reviewed by Western Michigan Homer Stryker MD School of Medicine , Kansas University, and PKD consortium IRB human research protection programs and determined to not be human subjects research.

Note that full information on the approval of the study protocol must also be provided in the manuscript.

Field-specific reporting

Please select the one below that is the best fit for your research. If you are not sure, read the appropriate sections before making your selection.

☒ Life sciences ☐ Behavioural & social sciences ☐ Ecological, evolutionary & environmental sciences

For a reference copy of the document with all sections, see nature.com/documents/nr-reporting-summary-flat.pdf

Life sciences study design

All studies must disclose on these points even when the disclosure is negative.

Sample size	ChIPs and RT-qPCR were performed using multiple independent cell cultures. Results were consistent and reproducible.
Data exclusions	No data was excluded that would change the interpretation of results.
Replication	ChIPs, qPCR were conducted with at least three replicates within an experiment, ChIPs results are from two or more independent assays.
Randomization	ChIPs and qPCR for human and mouse DNA were compared to each other.
Blinding	Experiments here are molecular in nature, so there was no blinding of samples.

Reporting for specific materials, systems and methods

We require information from authors about some types of materials, experimental systems and methods used in many studies. Here, indicate whether each material, system or method listed is relevant to your study. If you are not sure if a list item applies to your research, read the appropriate section before selecting a response.

Materials & experimental systems

n/a	Involved in the study
☐	☒ Antibodies
☐	☒ Eukaryotic cell lines
☒	☐ Palaeontology and archaeology
☒	☐ Animals and other organisms
☒	☐ Clinical data
☒	☐ Dual use research of concern
☒	☐ Plants

Methods

n/a	Involved in the study
☒	☐ ChIP-seq
☒	☐ Flow cytometry
☒	☐ MRI-based neuroimaging

Antibodies

Antibodies used	Anti-G4 DNA antibody clone BG4, Sigma MABE917, multiple lots were used (1/40 for ChIP, 1/50 for IF) and variability in G4 specificity was not observed, lot#s 303747, 3563069 , anti-phosphoserine139 histone H2AX, Novus Biologicals (NB 100-74435) lot# XH351179

	(used at 1/100), HRP-conjugated anti-rabbit IgG (Thermo Scientific, 65-6120, lot# QE215051 1/3000), Anti-FLAG tag, Cell Signaling, Cat # 14793S, lot# 7 used at 1/800. Anti-rabbit Alexa Fluor 568 (Invitrogen, A11011, lot# 2500544) was used at 1:1000. Anti-G4 nanobodies (SG4) were expressed from Addgene plasmids 196071 and 196072 and purified, used at 1/10 for dot blot, 1/50 for IF microscopy. Anti-RAD51 Novus Biologicals, (NB 100-148) lot# 45089 was used at 1/100. PC-1 antibody (used at 1/200 for Western) was provided by Dr. Chris Ward, University of Kansas Medical Center.
Validation	Antibodies were validated by the manufacturers: BG4 has specificity for G4 DNA but not non-G4 DNA judged by ELISA. Anti-gammaH2AX shows a single band by Western of the predicted size, Anti-FLAG antibody detected FLAG-tagged transgenes expressed in HEK293T by Western. Anti-RAD51 was validated by genetic strategies.<br><br>SG4 and SG4-R105 nanobody proteins were purified as described by Galli et al., 2022, and tested for G4 binding in both IF and dot blot with a G4-folded oligonucleotide (in manuscript). Both nanobodies were >95% pure as judged by SDS PAGE and performed as expected.

Eukaryotic cell lines

Policy information about cell lines and Sex and Gender in Research

Cell line source(s)	mIMCD-3 are mouse collecting duct renal cells that were purchased from the ATCC, cat# CRL-2123 lot#70008043. HEK293T cells were a gift from Dr. Tom Rothstein from the center for Immunobiology at Homer Stryker MD School of Medicine, Wmed. Transfer of ADPKD and normal human tissue for microscopy from the Polycystic Kidney Disease Research Resource Consortium was approved by the U24-CCS human material letter of transfer.
Authentication	ChIPs and expression experiments here only need cells of human and mouse origin, and for each to encode PKD1. Human PKD1 and PCNA (control) were amplified from HEK293 using human gene-specific (NCBI primer blast) primers. mIMCD-3 cells likewise expressed mouse Pkd1 and Pcna, judged by RT-PCR using cDNA-specific or genome-specific primers. RT-PCR and genomic amplicons obtained for both cell lines were validated and were of the expected size, correct origin (human or mouse), and sequence (NCBI blast).
Mycoplasma contamination	mIMCD-3 cells were tested for mycoplasma by ATCC, and tested negative.<br>HEK293T cells tested negative for mycoplasma.
Commonly misidentified lines<br>(See ICLAC register)	Human HEK293T is a commonly used cell line for transgene expression and other molecular studies, for the purposes here a cell line of human origin is needed for PKD1 PCR. Human-specific primers returned human PKD1 and PCNA PCR amplicons, as expected. mIMCD-3 is a commonly used in the kidney field for ADPKD research, PCR for mouse Pkd1 and Pcna verify both are in the genome, as expected.

Plants

Seed stocks	na
Novel plant genotypes	na
Authentication	na
